# Technological Feature Assessment of Lactic Acid Bacteria Isolated from Cricket Powder’s Spontaneous Fermentation as Potential Starters for Cricket-Wheat Bread Production

**DOI:** 10.3390/foods9091322

**Published:** 2020-09-19

**Authors:** Viola Galli, Manuel Venturi, Niccolò Pini, Lisa Granchi

**Affiliations:** 1 Department of Agriculture, Food, Environment and Forestry (DAGRI), University of Florence, Via San Bonaventura n. 13, 50145 Florence, Italy; viola.galli@unifi.it (V.G.); niccolo.pini@unifi.it (N.P.); lisa.granchi@unifi.it (L.G.); 2 FoodMicroTeam s.r.l., Via di Santo Spirito n. 14, 50125 Florence, Italy

**Keywords:** edible insects, sourdough, nutritional value, lactobacilli, baked products, spontaneous fermentation

## Abstract

The bacterial community profile of cricket powder highlighted the presence of four main genera: *Bacteroides* spp., *Parabacteroides* spp., *Lactococcus* spp., and *Enterococcus* spp. The spontaneous fermentation of cricket powder allowed for the isolation and characterization of seven lactic acid bacteria strains belonging to six species: *Latilactobacillus curvatus*, *Lactiplantibacillus plantarum*, *Latilactobacillus sakei*, *Lactococcus garvieae*, *Weissella confusa*, and *Enterococcus durans*. The strains were characterized and selected according to different technological properties. *L. plantarum* CR L1 and *L. curvatus* CR L13 showed the best performance in terms of general aminopeptidase activity, acidification, and growth rate in MRS broth and in dough with cricket powder and wheat flour, as well as robustness during consecutive backslopping. Thus, they were used as starter-mixed to produce sourdough to manufacture bread fortified with 20% cricket powder. The addition of cricket powder led to a significant increase of protein (up to 94%) and lipid content, from 0.7 up to 6 g/100 g of bread. Spontaneous fermentation represents a source of microbial diversity that can be exploited in order to obtain potential starters for food with innovative ingredients. Edible insects powder can be successfully added in leavened baked goods to enhance their nutritional value.

## 1. Introduction

Eating insects or foods containing insects is a common practice in many areas in Asia, Africa, and America [1,2]. The United Nations Food and Agriculture Organization (FAO) reported that more than 1900 species of insects, including meal larvae, crickets, ants, grasshoppers, and flies, are eaten worldwide [2]. Insects are a source of high-value and digestible protein with low environmental impact, so they might represent a cost-effective and more sustainable alternative to animal-based proteins in the human diet. Though considered food and subject to the European Union legislation on novel foods [3], insects and insect-derived products are not allowed for human consumption in all European countries [4]. According to some market studies, Europe is becoming the fastest growing market for edible insects [5], with an expected rapid increase of the demand, especially for cricket-based products [6]. Crickets (*Acheta domesticus*) are included by the European Food Safety Authority (EFSA) in the list of the insects with the biggest potential for food and feed [7]. They contain not only significant amounts of protein (which can reach up to 70 g/100 g dry matter), fat (especially polyunsaturated fatty acids), and fiber (due to the presence of chitin), but also of vitamins (mainly those of the B-group) and minerals (e.g., iron, selenium, and zinc) [2,8]. However, the insect introduction into Western diet is limited due to low consumer acceptance of insect-based foods, mainly because of their appearance [9], reducing their application as a potential ingredient for the development of food with improved nutritional features. To overcome this hurdle, different formulations for insect consumption have been suggested. Indeed, the degree of processing strongly affects the consumer acceptance towards insect food products. Formulations as powder, protein extracts, or protein hydrolysates have been shown to increase appreciation by consumers, reducing their negative perception [10,11]. Since the constant development of innovative baked goods with unconventional flours, edible insect powder might constitute a source of novel ingredients to be used in the bakery industry. These powders are obtained from reared crickets that are killed by a drying process and entirely ground. Several authors have reported the incorporation of cricket powder for bread production [12,13,14] and gluten-free bread [15,16] in order to enhance the nutritional value of bread and to evaluate the technological properties of cricket powder as alternative sources of protein. Many leavened baked goods are manufactured using sourdough. Sourdough is a complex ecosystem constituted by lactic acid bacteria and yeasts, exerting positive effects on the rheological, nutritional, sensory, and shelf-life properties of the final products [17]. Moreover, the use of sourdough for bread production is an additional source of potential innovation due to its large microbial biodiversity that is highly adapted to this specific environment and able to persist during consecutive backsloppings. In particular, lactic acid bacteria (LAB) are considered the main group responsible for these improved characteristics, thanks to their activities in the raw matrix and to their different metabolic capabilities [18,19], e.g., peptidase activities, acidification, exopolysaccharides production, and anti-nutritional compound reduction. The LAB of various genera have already been identified in cricket powder formulations [14,20,21] through culture-dependent and independent methods. Particularly, together with bacteria associated with the insect gut such as *Bacteroides* and *Parabacteroides* spp., the LAB of the genera *Enterococcus* spp., *Lactococcus* spp, *Lactobacillus* spp., and *Pedioccoccus* have been found. However, to the best of our knowledge, LAB isolation and identification have not been carried out during consecutive backsloppings of this novel ingredient. Therefore, the aim of this work was first to characterize cricket powder microbial community, and then to isolate LAB strains from a cricket powder’s spontaneous fermentation carried out for 10 days, thus identifying highly adapted strains for this specific matrix. The strains were evaluated for several pro-technological features in order to select the most suitable to be used as a potential starter in sourdough bread fortified with cricket powder.

## 2. Materials and Methods

### 2.1. Bacterial Community Profile of Cricket Powder

Cricket powder was provided by DL Novel Food Srl Bra Cuneo, Italy. 16S metagenetics were carried out at BMR Genomics (Padua, Italy) by using the Illumina MiSeq platform. The V3–V4 region of the 16S rRNA gene was amplified using the primers Pro341F and Pro805R [22] to assess the bacterial community. After the quality trimming and merging of raw data, filtered sequences were used for subsequent analysis.

### 2.2. Cricket Powder’s Spontaneous Fermentation

Initially, 50 g of cricket powder were mixed with tap water in a ratio of 1:1. The dough was incubated at 30 °C for 24 h and propagated (25% of the total dough *w*/*w*) for 10 days at the same time and temperature. At the end of each fermentation step, microorganism enumeration was carried out and pH was measured.

### 2.3. Microorganism Enumeration

Lactic acid bacteria, yeasts, Enterobacteriaceae, and total mesophilic aerobic populations were quantified, through serial dilutions, in cricket powder and dough samples. Ten gram of dough sample were homogenized with 90 mL-sterile saline solution. The diluted suspensions were plated on different culture media. For LAB enumeration, deMan Rogosa Sharpe (MRS) agar (Oxoid, Basingstoke, Hampshire, UK) was used. After 24–48 h of incubation at 30 °C in microaerophilic conditions in jars with the AnaeroGen enzymatic kit (Oxoid Ltd., Hampshire, UK), LAB colonies were counted. Yeasts, plated on MYPG (Malt, Yeast, Peptone, Glucose) agar containing sodium propionate (2 g/L), were enumerated after incubation for 48 h at 30 °C under aerobic conditions. Enterobacteriaceae were counted using a Violet Red Bile Glucose Agar (VRBGA) medium (Oxoid, Basingstoke, Hampshire, UK), and plates were incubated at 37 °C for 24 h. The total mesophilic aerobic bacteria were determined on Plate Count Agar (PCA, Oxoid Basingstoke, Hampshire, UK) at 30 °C for 48 h. Plate counts were performed in duplicate.

### 2.4. Monitoring of LAB Populations by RAPD-PCR Analysis

LAB populations, during the consecutive backsloppings in the cricket powder’s spontaneous fermentation and during the robustness test, were monitored through randomly amplified polymorphic DNA (RAPD) analysis. The DNA amplification was carried out as described by Venturi et al. [23] with the primers OPL-05 (5′ ACGCAGGCA 3′) [24], P1 (5′ ACGCGCCCT 3′) [25], and RD1 (5′ GCTTAAGGAGGTGATCCAGCC 3′) [26]. Amplification products were separated at 100 volts for 2.5 h on a 1.4% (*w*/*v*) agarose gel (Lonza Group Ltd., Basel, Switzerland) containing ethidium bromide (Sigma e Aldrich, St Louis, MO, USA) and a TEB buffer (1 M Tris, 10 mM EDTA, 0.9 M boric acid, pH 8.3). The profiles were captured after UV transillumination.

### 2.5. Genotypic Identification of Lactic Acid Bacteria

The lactic acid bacteria strains isolated from the cricket powder’s spontaneous fermentation were identified by 16S rRNA amplification and sequencing. DNA was extracted, and the 16S rRNA gene was amplified in a thermocycler (Techne LTD, Cambridge, UK) using the primers FD1 (5′ CAACAGAGTTTGATCCTGGCTCAG 3′) and RD1 (5′ GCTTAAGGAGGTGATCCAGCC 3′) [26]. Amplicons were purified using Nucleo Spin Extract II (Macherey-Nagel GmbH & Co. KG, Düren, Germany) and subjected to Sanger sequencing at BMR Genomics (Padua, Italy). The sequences obtained in the FASTA format were compared with those deposited in GenBank DNA database (http://www.ncbi.nlm.nih.gov/) using the basic BLAST search tools.

### 2.6. Lactic Acid Bacteria Technological Properties Characterization

#### 2.6.1. Peptidase Activity

LAB, grown overnight in an MRS broth, were counted by a Neubauer improved counting chamber (Marienfeld, Lauda-Königshofe, Germany) to obtain a cell suspension of 9 log cell/mL. Cells were harvested by centrifugation at 12,000× *g* for 5 min, washed twice with a sterile 50 mM potassium phosphate buffer at pH 7.0, and re-suspended in the same buffer. General aminopeptidase (PepNC), glutamyl aminopeptidase (PepA), and endopeptidase (PepO) activities were measured according to Macedo et al. [27] using Lys-p-nitroanilide (p-NA), Leu-p-NA, Pro-Glu-p-NA, and NCBZ-Gly-Gly-Leu-p-NA (Sigma-Aldrich, St Louis, MO, USA), respectively, as synthetic substrates. The release of p-NA was measured spectrophotometrically at 410 nm by comparing the data to a p-NA calibration curve according to the work of [28].

#### 2.6.2. Exopolysaccharide Synthesis

To investigate the synthesis of exopolysaccharide (EPS), the isolated LAB strains were inoculated on MRS agar supplemented with 2% of three carbohydrates: sucrose, glucose, or raffinose. EPS synthesis was visually assessed through the examination of slimy colonies production on the plates after 48 h of incubation at 30 °C.

#### 2.6.3. Lactic Acid Bacteria Growth and Acidification Kinetics

The isolated LAB strains, grown overnight in an MRS broth, were inoculated at a concentration of approximately log 7.7 CFU/g into the MRS broth medium and left to ferment 24 h at 30 °C. Tubes were filled with the medium in order to create microaerophilic conditions. Growth was monitored measuring culture absorbance at 600 nm every hour using a spectrophotometer. Acidification was determined by measuring the pH every hour.

#### 2.6.4. Cricket-Wheat Sourdough Acidification

LAB strains were used to prepare sourdoughs containing 80% wheat flour (type 00) and 20% cricket powder with a dough yield [(amount of flour + amount of water) × 100/(amount of flour)] of 154, with and without NaCl addition (2% *w*/*w*). For LAB inoculum, cultures were grown overnight in an MRS broth and estimated by a Neubauer improved counting chamber (Marienfeld, Lauda-Königshofe, Germany) to obtain an initial cell density in sourdough of approximately log 8.0 CFU/g. Successively, they were centrifuged (5000× *g* for 20 min), washed in physiological solution, and re-suspended in the tap water used for the preparation of the doughs. According to the commonly used backslopping conditions, fermentations were carried at 30 °C for 8 h. The pH was measured every hour, and the total titratable acidity and LAB concentrations were determined after 8 h.

#### 2.6.5. Lactic Acid Bacteria Strain Robustness

Initially, a sourdough comprising 80% wheat flour (type 00) and 20% cricket powder with a dough yield of 154 was prepared. LAB were counted by a Neubauer improved counting chamber (Marienfeld, Lauda-Königshofe, Germany) to obtain an initial cell density in sourdough of about 7.3 log CFU/g and were simultaneously inoculated into the dough. The sourdough was backslopped ten times (25% *w*/*w*) and incubated at 30 °C. At the end of every fermentation step, microorganism enumeration was carried out on MRS agar. In order to monitor the strain trends during propagation, at least 20 colonies of the beginning of the experiment and at the end of the 4th, 7th, and 10th backsloppings were randomly selected from the plates containing the two highest sample dilutions and then were analyzed by RAPD-PCR to determine the percentage distribution of the inoculated LAB.

### 2.7. Determination of pH, TTA, and Volume

The pH values of the sourdoughs were determined by a pH meter (Metrohm Italiana Srl, Varese, Italy). The total titratable acidity (TTA) was calculated by weighing 10 g of dough samples and homogenizing them with 90 mL of distilled water for 3 min; the TTA was then expressed as the amount (mL) of 0.1 N NaOH to achieve a pH of 8.5. The increase of volume was assessed immediately and after 2 h of fermentation at 30 °C by placing 100 g of dough in a graduated cylinder (1 L). The volume increase was calculated using the following formula: [(*V*_2_ − *V*_0_)/*V*_0_] × 100, where *V*_2_ was the volume after the 2 h fermentation and *V*_0_ was the initial volume.

### 2.8. Determination of Organic Acids

For the organic acid determination, the sourdough samples were diluted ten times with distilled water and then filtered by Amicon^®^ Ultra-4 Centrifugal Filters (3000 Da NMWL) (Merck Millipore, Burlington, MA, USA) before the injection. Organic acid determination was carried out by HPLC analysis (Varian Inc., Palo Alto, CA, USA) following [29]. Data were collected and analyzed by using the Galaxie software (version 1.9.302.530, Varian Inc., Palo Alto, CA, USA). The fermentation quotient (FQ—the molar ratio between lactic and acetic acids) was calculated.

### 2.9. Bread Preparation

Three breads were prepared according to recipes reported in Table 1.

The sourdough was prepared by inoculating the strains that showed promising technological traits at an initial cell density of approximately 7 log CFU/g of dough. The dough was fermented at 30 °C for 18 h before its utilization as ingredient for SDBread (bread produced by sourdough and only wheat flour) and CSDBread (bread produced by sourdough and wheat flour and cricket powder). SDBread was prepared with type “00” wheat (*Triticum aestivum* L.) flour (COOP, Casalecchio di Reno, Bologna, Italy; protein content: 12.5 g/100 g), while CSDBread and CBYBread (bread produced by baker’s yeast and wheat flour and cricket powder) were incorporated with 20% of cricket powder. The CBYBread was fermented by using only baker’s yeast (Zeus Iba, Florence, Italy). The ingredients were added at the same time and mixed for 10 min in a model RS12 twin arms mixer (Bernardi, Cuneo, Italy). The doughs were placed on trays at 30 °C with 88–90% relative humidity in a Unipan proofing chamber (Alaska, Costa di Rovigo, Rovigo, Italy) for 2 h. Samples were taken at the beginning and end of the leavening time. Finally, doughs were baked at 180 °C for 25 min in an oven (Rossella, Unox, Padua, Italy).

### 2.10. Approximate Chemical Composition of Breads

The approximate chemical composition of breads was assessed. Crude protein content was determined following by the methods of Lowry et al. [30]. Carbohydrate and lipid contents were determined following the work of Dubois et al. [31] and Marsh and Weinstein [32], respectively.

### 2.11. Statistical Analysis

The results are presented as the mean values ± standard deviation of two or three separate experiments for each test. The level of statistical significance was determined using one-way ANOVA (for multiple groups) followed by Tukey’s Test or Student’s *t*-test (for comparisons between two groups) (GraphPad Prism 6 software package). A *p* value of <0.05 was considered to be significant. The kinetics of microbial growth and acidification were modelled in agreement with the Gompertz equation using GraphPad Prism 6 (GraphPad Software, San Diego, CA, USA)

## 3. Results

### 3.1. Bacterial Community of Cricket Powder

**Figure 1:**
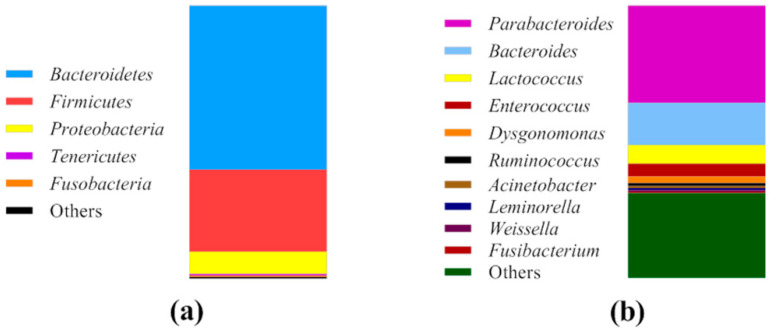
Relative abundance (%) of the phyla (**a**) and genera (**b**) present in cricket powder.

The results of cricket powder microbial diversity, assessed by the sequencing of the V3–V4 region of the 16S rRNA gene, are shown in Figure 1.

A total of 57,811 reads passed the filters applied in the QIIME split_library.py script. Most of the operational taxonomic units (OTU) were identified at the order level as 96.7%, while at the genus level, sequences were identified at 69.0%. Cricket powder was dominated by three bacterial phyla—Bacteroidetes (60.1% of all the sequences), Firmicutes (30.0%), and Proteobacteria (8.2%). Tenericutes and Fusobacteria were also detected in low abundance at less than 1%. Within Bacteroidetes, *Parabacteroides* spp. and *Bacteroides* spp. were the dominant genera at 35.6% and 15.5%, respectively. Three genera were identified among Firmicutes: The genus *Lactococcus* spp. was found at the highest relative abundance, accounting for 6.9%, followed by *Enterococcus* spp. (4.7%) and *Weissella* spp. (0.6%). *Leminorella* spp. and *Acinetobacter* spp., belonging to Proteobacteria, were found in low abundance at less than 1%.

### 3.2. Cricket Powder and Spontaneous Fermentation Microbiological Analysis

**Figure 2:**
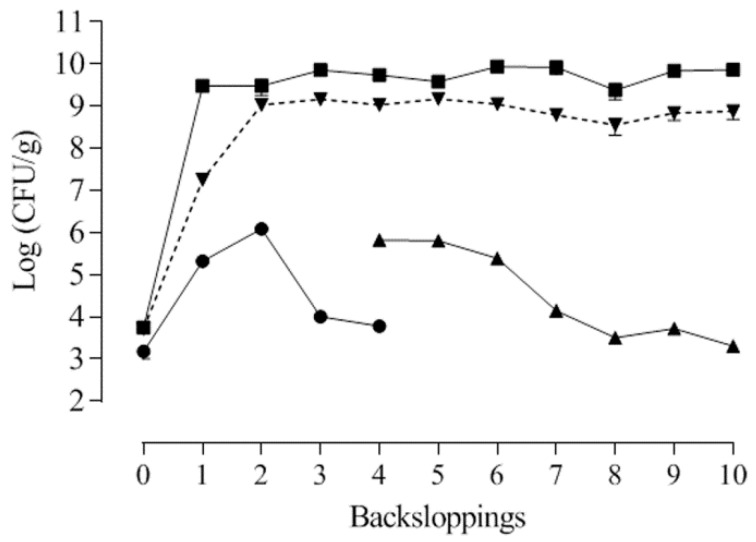
Lactic acid bacteria (dotted line), Enterobacteriaceae (triangle), yeasts (round), and total mesophilic count (square) concentrations (mean ± standard deviation) during the ten consecutive backsloppings in the spontaneous cricket powder fermentation.

Microbiological analyses indicated that cricket powder was characterized by cell densities of 3.71 ± 0.27 log CFU/g of LAB, of 3.51 ± 0.27 log CFU/g of yeasts, and of 4.12 ± 0.28 log CFU/g of the total mesophilic bacteria. Enterobacteriaceae were below the detection limit. In order to isolate indigenous LAB, a dough was prepared using cricket powder. The dough was backslopped daily ten times. At the end of each backslopping, LAB, yeasts, Enterobacteriaceae, and the total mesophilic bacteria were enumerated (Figure 2).

At the beginning, the microbial populations of the dough included 3.40 log CFU/g of LAB, 3.74 log CFU/g of the total mesophilic bacteria, and 3.17 CFU/g of yeasts. Enterobacteriaceae were below the detection limit. During the consecutive backsloppings the yeast population increased, reaching the maximum concentration of 6.08 log CFU/g at the end of the second day; then, it decreased, and yeasts were not detected anymore after the fourth backslopping. Unexpectedly, starting from the fourth backslopping, Enterobacteriaceae were detectable, attaining a value of approximately 5 log CFU/g. After the seventh propagation step, the population started decreasing, reaching a concentration of 3.30 log CFU/g at the end of dough propagation. The total mesophilic bacteria rapidly increased during the propagation, and they were found in concentration of 9.48 log CFU/g after the first 24 h of fermentation. The cell densities remained basically stable until the end of propagation.

The LAB cell density after the first day of propagation was 7.25 log CFU/g. Over the successive backsloppings, their number increased, reaching concentrations higher than 9 log CFU/g and then remaining stable until the end. The initial pH of the dough was 6.38, and it remained stable at about 6.5 during all the propagation steps despite the high LAB concentration.

### 3.3. Lactic Acid Bacteria Identification and Intraspecific Characterization

A total of 60 isolates of presumptive lactic acid bacteria, isolated on each day of propagation, were subjected to RAPD-PCR. The analysis allowed us to identify seven patterns named CR L1, CR L2, CR L13, CR L14, CR L15, CR L31, and CR L36 (Figure S1). The amplification and sequencing of the DNA from these isolates indicated that they belonged to six species: *Lactiplantibacillus plantarum* (formerly known as *Lactobacillus plantarum* [33]) CR L1, *Weissella confusa* CR L2, *Latilactobacillus curvatus* (formerly known as *Lactobacillus curvatus*) CR L13, *Lactococcus garvieae* CR L14 and CR L31, *Latilactobacillus sakei* (formerly known as *Lactobacillus sakei*) CR L15, and *Enterococcus durans* CR L36.

### 3.4. Lactic Acid Bacteria Technological Properties

The isolated lactic acid bacteria LAB strains were evaluated for their technological features.

#### 3.4.1. Peptidase Activity

The PepNC, PepA, and PepO activities of the strains were determined using Leu-p-NA, Glu-p-NA, and NCBZ-Gly-Gly-Leu-p-NA as substrates. The highest variability and values were found for PepNC activity (Figure S2). The values ranged from 0.25 ± 0.09 (*W. confusa* CR L2) to 2.17 ± 0.23 mMol/L of p-NA reached by *L. garvieae* CR L14, which was not statistically different from the enzymatic activities of *L. curvatus* CR L13 and *L. garvieae* CR L31. PepA and PepO activities were low in all the LAB strains when compared to PepNC activities, not exceeding 0.26 mMol/L of released p-NA. No significant differences were detected among the strains.

#### 3.4.2. Exopolysaccharide Production

The *W. confusa* CR L12 strain was the only LAB that showed a notable ability to produce EPS from sucrose after 48 h of incubation at 30 °C. No production was observed in the other six strains.

#### 3.4.3. LAB Growth and Acidification Kinetics

The LAB growth and acidification kinetics in the MRS broth at 30 °C were monitored over 24 h (Figure S3), and data were modeled according to the Gompertz equation (Table 2).

The goodness of fit of this model was appropriate for all the tested strains, with *R^2^* values higher than 0.90 (data not shown). *L. plantarum* CR L1 showed the best performance of growth; indeed, it displayed the highest maximum yield (C) of 1.56 ± 0.03 and the highest maximum rate (µ max) of 0.29 ± 0.02, along with a short lag phase. The lowest growth was observed for both *L. garvieae* strains (less than 0.6), while the lowest µ max was displayed by *L. curvatus* CR L13. *L. garvieae* CR L31 showed the longest lag phase. All the LAB reached the stationary phase after approximately 20 h. The LAB acidification ability was assessed by measuring the pH for 24 h. The kinetic parameters obtained through the Gompertz equation were used to describe the potential acidifying performances of the tested strains. *L. plantarum* CR L1 proved to be the most acidifying strain, reaching a final pH value of 3.78 after 24 h and hence having the highest maximum yield (C) value. *W. confusa* CR L2, *L. curvatus* CR L13, *L. sakei* CR L15, and *E. durans* CR L36 acidified the MRS broth to values below 4.5, while the two *L. garvieae* strains final pH was approximately 4.5. However, the highest µ max was displayed by *L. garvieae* CR L31 at higher than 0.3 dpHh^−1^, and this strain also showed the longest lag phase.

#### 3.4.4. Effect of Salt on Wheat-Cricket Powder Sourdough Acidification and Cell Concentrations

**Figure 3:**
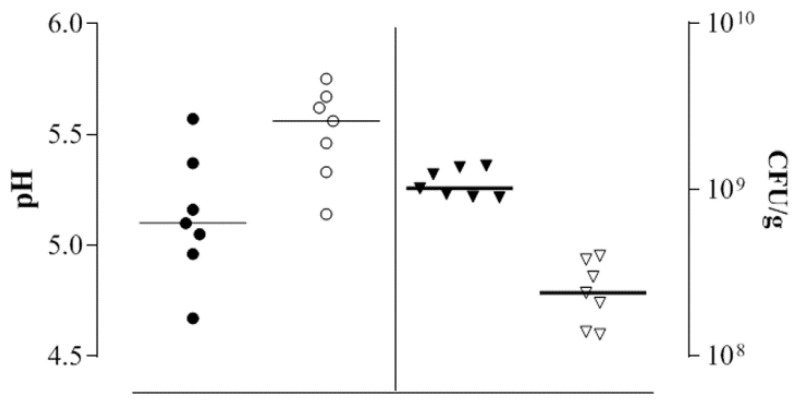
Scatterplot of the final pH and cell concentrations of the doughs with NaCl addition (black symbols) and without NaCl addition (white symbols), inoculated with the 7 strains after 8 h of fermentation at 30 °C. pH values are represented by circles plotted on the left axis; bacterial cell densities are represented by triangles plotted on the right side. Horizontal bars represent the median values.

In order to investigate the influence of salt addition on the acidification performances and on cell densities, each strain was inoculated in a sourdough made with 80% wheat flour and 20% cricket powder with and without NaCl (2% *w*/*w*) addition. The scatterplot (Figure 3) represents the pH value and cell density distributions after 8 h of fermentation.

In all the sourdoughs, the addition of salt reduced the acidification. The pH median value of the dough without salt was 5.10, while this value was 5.56 in doughs containing NaCl. *L. plantarum* CR L1 was the most acidifying strain, reaching final pH values of 4.67 and 5.14 with and without salt, respectively. The median values of the ∆pH (data not shown) were 0.94 without NaCl and 0.40 with NaCl. *L. plantarum* CR L1, *W. confusa* CR L2, *L. curvatus* CR L13, and *E. durans* CR L36 reached a concentration of 9 log CFU/g, while the other strains reached a concentration of around 8.9 log CFU/g. The supplement of NaCl negatively influenced the final cell concentrations. In the presence of salt, none of the strains reached 9 log CFU/g; the highest concentration in the dough with NaCl was achieved by *L. plantarum* CR L1 at 8.6 log CFU/g, followed by *L. curvatus* CR L13 and *E. durans* CR L36.

#### 3.4.5. Lactic Acid Bacteria Robustness during Consecutive Backsloppings

**Figure 4:**
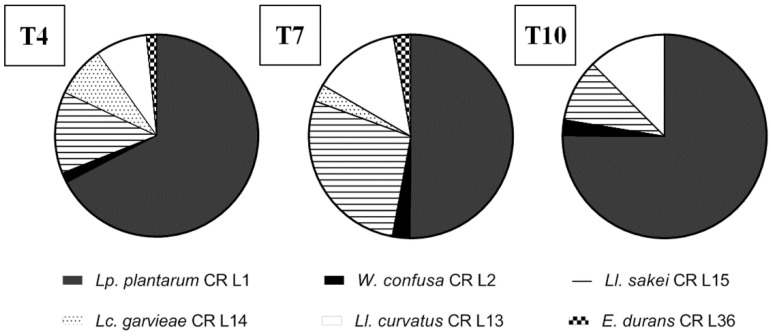
Percentage distribution of the 7 lactic acid bacteria strains at the end of the 4th (T4), 7th (T7), and 10th (T10) backslopping in wheat flour and cricket powder sourdough.

The strain robustness (the ability to dominate and persist during consecutive backsloppings) was evaluated by co-inoculating all the strains at the initial concentration of 7.3 log CFU/g of dough. Soon after the first day of backslopping, the total LAB concentration was attained at 9.78 log CFU/g, which remained stable until the end. After the fourth, seventh, and tenth backslopping, 20 colonies were subjected to RAPD-PCR in order to monitor the strain percentages. As shown in Figure 4, after the fourth backslopping, 67% of the isolates were *L. plantarum* CR L1, followed by *L. sakei* CR L15 (13%); *L. garvieae* CR L31 was not detected.

Our results showed that the proportions among the strains remained similar between the fourth and seventh refreshments. Particularly, *L. plantarum* CR L31 was present at the highest percentage (50% of the isolates), followed by *L. sakei* CR L15 (28%). At the end of the propagation, *L. plantarum* CR L1, represented the majority of the isolates, 75%, followed by *L. curvatus* CR L13 (12%), *L. sakei* CR L15 (10%), and, in the lowest concentration, *W. confusa* CR L2 (2.5%). The *E. durans* strain and both *L. garvieae* strains were not detected, thus showing the lowest robustness.

### 3.5. Dough Fermentation and Bread Making

**Figure 5:**
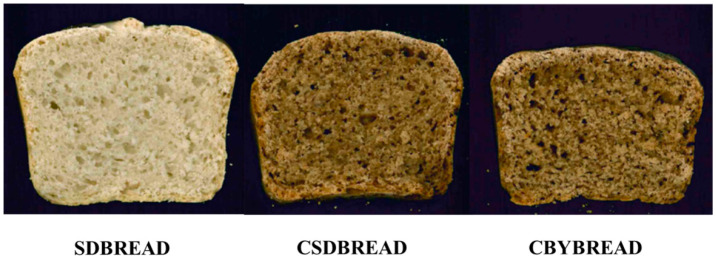
Slices obtained from the loaves of experimental breads.

The results of the acidification, volume increase, and microorganism concentration of the doughs prepared with the use of only wheat flour and sourdough (SD), the addition of cricket powder and the use of sourdough (CSD), and the addition of cricket powder and use of only baker’s yeast (CBY) are reported in Table S1. *L. plantarum* CR L1 and *L. curvatus* CR L13, which were selected for their technological traits, were inoculated for the production of sourdough for bread manufacturing. At the end of fermentation, the SD dough reached a pH below 4.5, while the final pH of the CSD dough was higher at 5.15. The initial pH of the CSD dough was higher due to the buffering capacity of the cricket powder; indeed, the pH decrease was not statistically different between the samples, remaining at around 0.47. The final total titratable acidity values were higher in the CSD and CBY doughs, while the SD dough displayed the lowest value. This difference depended on the incorporation of cricket powder into the dough since the control bread without sourdough inoculum also displayed a considerable TTA value of 7.6 mL. Cricket powder negatively affected the volume increase; indeed, SD dough showed the highest final volume, as seen Figure 5 where slices of experimental breads are shown.

Regarding organic acid production, no difference in lactic acid concentration was observed between the two SD doughs, while the highest content of acetic acid was displayed in the CSD dough, thus leading to a different fermentation quotient. The microbiological analyses did not show any significant differences in the LAB and yeast concentrations, with values of about 8.6 and 7.8 log CFU/g, respectively.

### 3.6. Biochemical Characterization of the Breads

Table 3 presents the approximate chemical composition of breads.

The addition of cricket powder in the formulation significantly increased the protein content of the breads by +82% and +94% in the CSDBread and CBYBread, respectively, compared to the SDBread. Lipid content was also affected by cricket powder addition, as both the CBreads presented a significantly higher content than the SDBread at more than 5.5%. The amount of carbohydrates was higher in breads without cricket powder due to the higher content of carbohydrates in wheat flour compared to cricket powder. In terms of moisture, no significant differences were found among the samples.

## 4. Discussion

**Figure 6:**
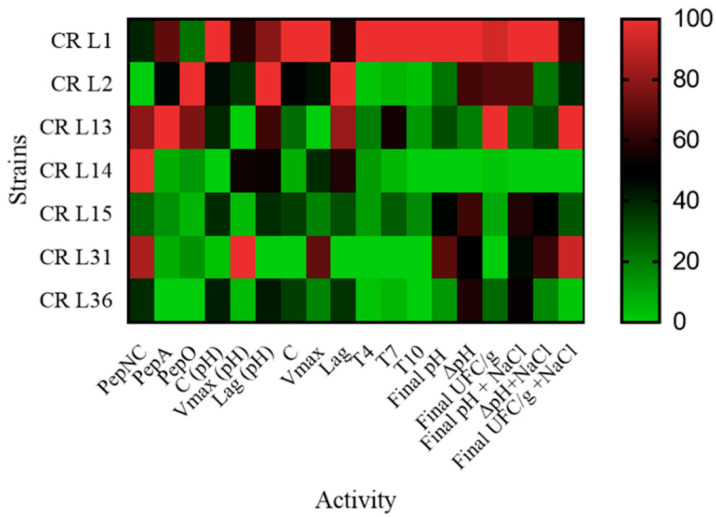
Heatmap based on lactic acid bacteria strains technological features assessed in the screening: peptidase activities (general aminopeptidase (PepNC), glutamyl aminopeptidase (PepA), and endopeptidase (PepO)); acidification and growth parameters (C, Vmax, and Lag) in an MRS medium; pH; ΔpH (difference between final and initial pH); cell concentrations (CFU/g) and Δcell concentrations (difference between final and initial cell concentration; and CFU/g) in doughs with NaCl addition and without NaCl addition; percentage of bacterial strains at the end of 4th (T4), 7th (T7), and 10th (T10) backslopping in the robustness test. The values of the activities were standardized to range from 0 to 100. Colors correspond to normalized mean data levels from low (green) to high (red).

In the first part of the work, a bacterial characterization of cricket powder was carried out through a next generation sequencing (NGS) analysis that allowed for an in-depth evaluation of the occurring bacteria. In accordance with the findings of other authors [14,20,21], the taxonomic analysis revealed the presence of three dominant phyla: Bacteroidetes, Firmicutes, and Proteobacteria. Bacteroidetes includes species associated with the gut microbiota of human and insects [34,35] that were probably transferred from intestinal tracts when the crickets were crushed into powder. Here, Firmicutes comprised *Clostridiales* and *Lactobacillales* of the genera *Lactococcus* spp., *Enterococcus* spp., and *Weissella* spp., all of which are of interest in food fermentation. As reported by Cappelli et al. [36], the microbiological risk of insect consumption has to be taken into account, and it is strictly correlated to the species, rearing, transformation and type of consumption. Even though a large biodiversity is found in cricket powder, the presence of pathogenic bacteria was not highlighted by metagenomics analysis. Further investigations were not performed because of the cooking of the final products. Cricket powder was used to carry out a spontaneous fermentation in order to isolate LAB strains that were adapted and able to compete and persist in this food matrix. The propagation over ten days allowed for the establishment of a complex microbiota consisting of LAB, yeasts, and Enterobacteriaceae. LAB were present at high concentrations throughout the propagation. A total of seven strains belonging to six species were identified throughout the propagation via RAPD-PCR and 16S rRNA sequencing, revealing the presence of three species that belonged to the genera already determined through NGS analysis: *Enterococcus durans*, *Lactococcus garvieae*, and *Weissella confusa*. *Enterococcus durans* has been frequently isolated in several dairy products made with pasteurized and raw milk, and it can occur as natural starter cultures during cheese making [37,38]. *Weissella confusa* has been frequently detected in the spontaneous fermentation of fish products and starchy- or cereal-based foods [39], as well as in dried locust [14]. *Lactococcus garvieae* is an emerging zoonotic agent isolated from fish and has been the most frequently species isolated from insect frass samples and cricket powder [20,40]. It has also been found in the spontaneous fermentation of chickpea [41], wheat grain, and quinoa flours [42]. The spontaneous fermentation of unconventional flours has often led to the selection of species that are not considered endemic to traditional sourdough and that are dominated by *Lactobacilli*. Nevertheless, four species were previously isolated in cereal sourdoughs. *Lactiplantibacillus plantarum* is often isolated in different ecosystems due to its versatile metabolism [43]. *Latilactobacillus curvatus* is frequently isolated in association with other *Lactobacilli* (particularly *Latilactobacillus sakei*) in fermented meat, such as sausage [44], and during the fermentation of kimchi [45]. The disappearing of yeasts during spontaneous fermentation has already been observed in the spontaneous fermentation of legumes and pseudocereals [46,47,48,49]. Enterobacteriaceae probably developed due to external contamination, and it survived during all the propagation because of the relatively high pH of the dough. A characterization of the seven identified strains based on technological metabolic traits (Figure 6) was carried out.

The direct selection of starter microorganisms from the substrate to be fermented is considered an important feature in guaranteeing the rapid adaptation, intense acidification, and positive influence on the nutritional and technological properties of a specific matrix [50,51]. The peptidase activity of LAB, which is a strain-dependent property [52,53], is responsible for the production of peptides and amino acids that affect bread quality as taste-active, flavor precursors, or bioactive compounds [54]. Among the three tested peptidase activities, our results showed that PepA and PepO activities were very low in all the LAB strains when compared to PepNC enzymatic activities, as reported in other papers [28,55]. The two *L. garvieae* strains displayed the highest values of PepNC, which were not statistically different from the enzymatic activities of *L. curvatus* CR L13. The EPS production was also tested, since microbial EPS can improve the rheology and texture of gluten-free or low-gluten dough [56]. Only the *W. confusa* CR L2 strain was able to produce EPS from sucrose, corroborating that the ability to produce these polymers is one of the distinctive phenotypic features of the genus *Weissella*. Acidification and growth capabilities are essential characteristics for a microbial starter to be used in sourdough fermentation. A short lag phase, a high acidification rate, and a high yield are of particular interest. In our experiment, the kinetic parameters obtained through the measurements of pH and growth over 24 h indicated that the *L. plantarum* CR L1 strain was the best performing, showing the highest and fastest acidification both in the MRS medium and the sourdough made of 20% cricket powder. The acidification in cricket-wheat sourdough was also evaluated with the addition of 2% of salt, which is a common ingredient in bread and cereal-based products. As expected, the addition of salt worsened the acidification and growth parameters. Together with *L. plantarum* CR L1, *L. curvatus* CR L13 showed good performance in the presence of salt. In this regard, homofermentative LAB and facultative heterofermentative LAB such as *L. plantarum* and *L. curvatus* are considerably more resistant to high NaCl levels than heterofermentative species [57], and *L. plantarum* has been reported to grow at 8% NaCl [58,59]. Finally, the robustness of the strains was assessed. The intrinsic robustness of microorganisms is one of the parameters that have to be considered in order to select a suitable starter. The ability to persist and dominate during consecutive backsloppings is of particular industrial interest and contributes to sourdough stability [60]. Usually, four-to-seven backsloppings are required to reach microbial stability (a stable proportion among the microorganism population) [61,62]. The dominance of *L. plantarum* CR L1 was already observed at the fourth backslopping and was retained until the end of propagation. *L. curvatus* CR L13 microbial population represented the second-highest percentage of LAB at the end of propagation, showing an increase of its prevalence during the consecutive backsloppings. Based on the obtained results, *L. plantarum* CR L1 and *L. curvatus* CR L13 were selected as starters for the production of sourdough for bread manufacturing. Both the doughs with cricket powder were characterized by a higher TTA than the SD dough, as found by Osimani et al. [63]. This finding was probably due to the high ash content of cricket powder that affects a dough’s buffering capacity. As mentioned above, the integration of insect powder in baked goods represents a possible strategy to increase their nutritional quality, though changes in the dough structure and rheology of the doughs have to be taken into account. Substitution with cricket powder has been shown to increase dough stability, reduce the degree of softening, and lower water adsorption [12,13,63]. As expected, the inclusion of cricket powder negatively influenced the volume increase independently of the leavening agent, though it remained acceptable. The addition of cricket powder significantly increased the total protein content to more than 80% compared to bread with only wheat flour, thus increasing the overall nutritional quality of the breads. In addition to the protein content increase, breads with cricket powder showed a higher level of fat compared than the SD breads. Cricket powder contains high levels of saturated fatty acids (mainly C16:0 and C18:0) and monounsaturated fatty acids (mainly C18:1 n−9), while wheat flour includes mostly poly unsaturated fatty acids (mainly linoleic and linolenic acids). leading to different nutritional qualities and sensitivities to oxidation during storage [63].

## 5. Conclusions

The microbiota of cricket powder were characterized by several gut-associated bacteria and, among lactic acid bacteria, by the *Lactococcus* spp., *Enterococcus* spp., and *Weissella* spp. genera. The spontaneous fermentation of cricket powder, monitored through a culture-dependent method, allowed for the development of seven lactic acid bacteria species that are highly adapted to this ecosystem. The technological characterization of the indigenous LAB led to the selection of two suitable strains, *L. plantarum* CR L1 and *L. curvatus* CR L13, to be used for the manufacturing of cricket-wheat-based sourdough products. Despite the negative effect on bread volume, cricket powder replacement provided higher protein and fat contents than those of bread with only wheat flour. Overall, these results highlighted the good suitability of the microbial starter for the production of cricket-wheat flour bread with improved nutritional quality. Further studies should be carried out in order to improve consumer acceptance, test the selected LAB in other products, and optimize the final product formulations. Nevertheless, the combination of lactic acid bacteria fermentation and the use of edible insects as alternative flour can lead to the manufacturing of baked goods with improved nutritional traits that meet the demand of more sustainable food.

## Figures and Tables

**Table 1:** Ingredients and fermentation conditions (time and temperature) for sourdough preparation, bread preparation, and dough yield. SDBread: bread produced by sourdough and only wheat flour; CSDBread: bread produced by sourdough and wheat flour and cricket powder; and CBYBread: bread produced by baker’s yeast and wheat flour and cricket powder.

	Ingredients	SDBread	CSDBread	CBYBread
Sourdough 18 h at 30 °C	Wheat flour (g)	162.5	162.5	--
	Water (mL)	87.5	87.5	--
	*L. plantarum* CR L1 *L. curvatus* CR L 13 7 log (CFU/g)	7	7	--
Breads 2 h at 30 °C	Sourdough (g)	250	250	
	Wheat flour (g)	474.2	347.5	510.0
	Cricket powder (g)	--	126.7	126.7
	Water (mL)	275.8	275.8	363.3
	Salt (g)	9	9	9
	Baker’s yeast(g)	9	9	9
Dough yield		157	157	157

**Table 2:** Kinetic parameters of lactic acid bacteria growth and acidification in an MRS broth, as modelled according to the Gompertz equation. C: maximum yield; µ max: maximum rate; Lag: length of the lag phase.

Strains	Growth Kinetic Parameters		pH Kinetic Parameters		
	µ Max	Lag (h)	C	µ Max (-dpHh^−1^)	Lag (h)
*L. plantarum* CR L1	0.29 ± 0.02 ^d^	6.92 ± 0.2 ^bc^	2.06 ± 0.05 ^c^	0.28 ± 0.02 ^bc^	5.77 ± 0.3 ^ab^
*W. confusa* CR L2	0.18 ± 0.01 ^bc^	5.79 ± 0.2 ^a^	1.63 ± 0.04 ^b^	0.23 ± 0.02 ^ab^	5.16 ± 0.3 ^a^
*L. curvatus* CR L13	0.08 ± 0.01 ^a^	6.11 ± 0.4 ^ab^	1.59 ± 0.07 ^b^	0.15 ± 0.01 ^a^	6.19 ± 0.6 ^ab^
*L. garvieae* CR L14	0.17 ± 0.02 ^bc^	6.94 ± 0.2 ^bc^	1.28 ± 0.04 ^a^	0.27 ± 0.04 ^b^	6.46 ± 0.3 ^abc^
*L. sakei* CR L15	0.12 ± 0.01 ^ab^	7.69 ± 0.2 ^cd^	1.58 ± 0.07 ^b^	0.16 ± 0.02 ^a^	6.92 ± 0.2 ^bc^
*L. garvieae* CR L31	0.24 ± 0.04 ^cd^	8.52 ± 0.1 ^d^	1.30 ± 0.03 ^a^	0.37 ± 0.04 ^c^	7.99 ± 0.2 ^c^
*E. durans* CR L36	0.11 ± 0.01 ^ab^	7.21 ± 0.4 ^bc^	1.60 ± 0.08 ^b^	0.16 ± 0.02 ^a^	6.80 ± 0.6 ^abc^

Values in the same column with different letters (a–d) are significantly different (*p* < 0.05). The data are the means of three independent experiments ± standard deviations.

**Table 3:** Approximate chemical composition of breads (g/100 g).

	Protein	Carbohydrates	Lipids	Dry Matter
**SDBread**	12.6 ± 0.8 ^a^	75.3 ± 2.1 ^b^	0.7 ± 0.1 ^a^	69.6 ± 0.42 ^a^
**CSDBread**	22.9 ± 1.1 ^b^	61.6 ± 3.4 ^a^	5.8 ± 0.9 ^b^	70.1 ± 0.52 ^a^
**CBYBread**	24.5 ± 1.6 ^b^	60.9 ± 2.8 ^a^	6.0 ± 0.8 ^b^	69.4 ± 0.43 ^a^

Values in the same column with different letters (a, b) are significantly different (*p* < 0.05).

## Supplementary Materials

The following are available online at https://www.mdpi.com/2304-8158/9/9/1322/s1: Figure S1: Randomly amplified polymorphic DNA-polymerase chain reaction (RAPD-PCR) patterns; Figure S2: LAB peptidase activities; Figure S3: Growth and pH kinetics of lactic acid bacteria in the MRS broth; and Table S1: Acidification parameters, volume increase, and microorganism concentrations of the doughs at the end of fermentation.
