# Supplementary material for: Technological Feature Assessment of Lactic Acid Bacteria Isolated from Cricket Powder’s Spontaneous Fermentation as Potential Starters for Cricket-Wheat Bread Production
Source: Foods. 2020 Sep 19;9(9):1322. doi: 10.3390/foods9091322 (PMC7555194; doi:10.3390/foods9091322)
Supplement: Supplementary file 1 [file foods-09-01322-s001.pdf]

Supplementary

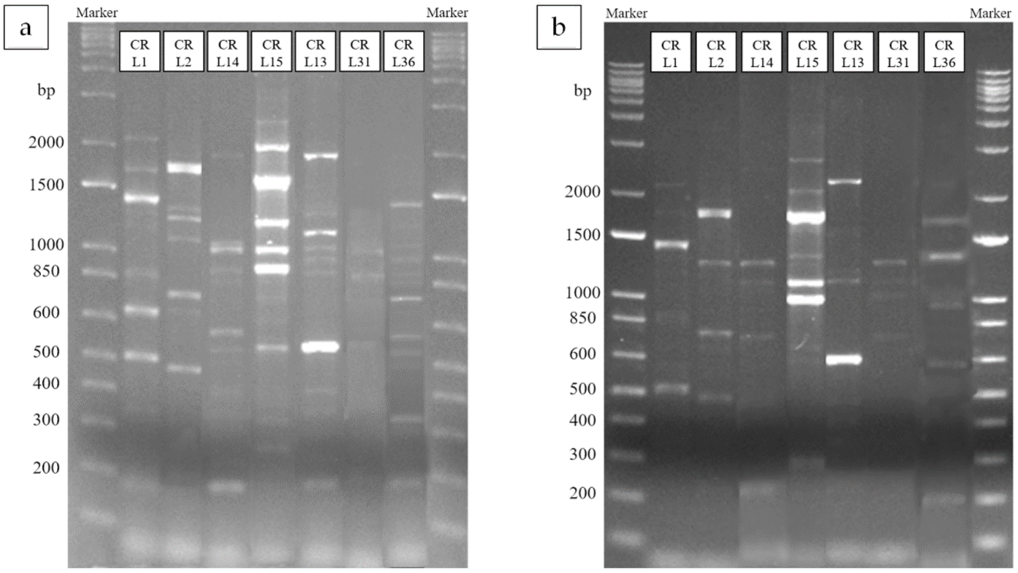

Figure S1. Randomly Amplified Polymorphic DNA-Polymerase Chain Reaction (RAPD-PCR) patterns of *Lp. plantarum* CR L1, *W.confusa* CR L2, *Lc. garvieae* CR L14, *Ll. sakei* CR L15, *Ll. curvatus* CR L13, *Lc. garvieae* CR L31, *E. durans* CR L36, obtained by using primer – along with RD1(a) and primer – along with RD1(b); Marker L100 DNA ladder.

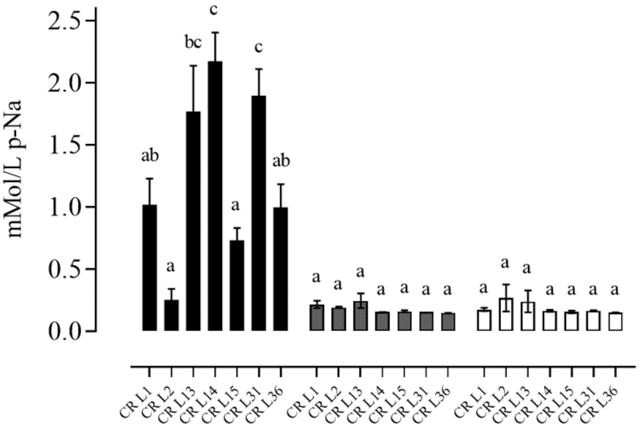

Figure S2. LAB peptidase activities, PepNC (black bars), PepA (grey bars) and PepO (white bars). Different letters (a–d) indicate statistically significant differences ($p<0.05$). The data are the means of three independent experiments (mean ± standard deviations).

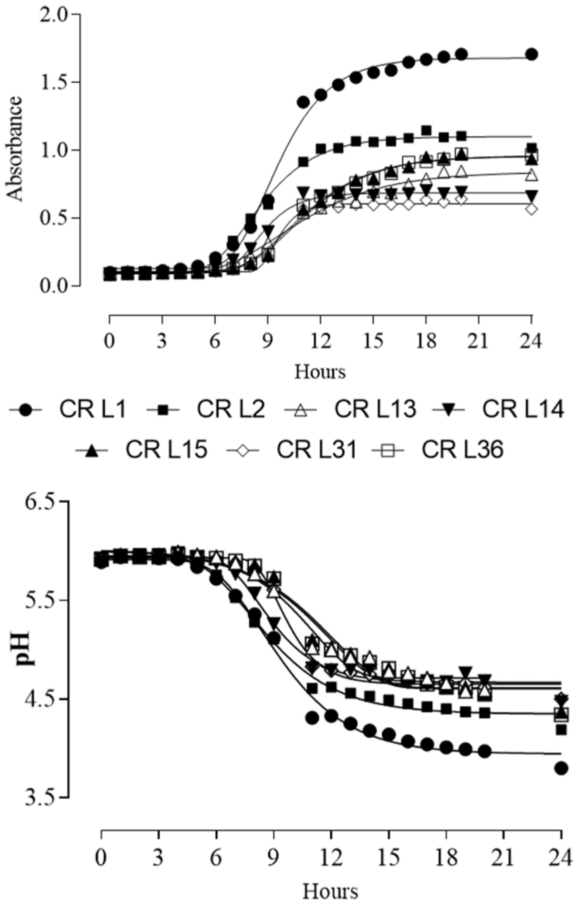

Figure S3. Growth (upper graph) and pH (lower graph) kinetics of lactic acid bacteria in MRS broth monitored for 24 hour at 30°C.

Table S1. Acidification parameters (pH and Total Titratable Acidity, TTA, organic acids), volume increase (%, ΔV/V0), microorganism concentrations (LAB and yeast) of the doughs at the end of fermentation; Δ: difference between the final and the initial value). Results are expressed as average ± standard deviation (n = 3).

	**Final pH**	**ΔpH**	**Final TTA (mL)**	**$\Delta V/V_{in}$ x 100**	**Lactic acid (g/100 g)**	**Acetic acid (g/100 g)**	**QF**	**LAB (CFU/g)**	**Yeast (CFU/g)**
**SD**	4.46 ± 0.06 ^{a}	−0.44 ± 0.05 ^{a}	2.95 ± 0.35 ^{a}	144 ± 10 ^{b}	0.19 ± 0.03 ^{a}	0.02 ± 0.0 ^{a}	6.33	(4.90 ± 0.78) × $10^{8\,a}$	(6.40 ± 0.22) × $10^{7\,a}$
**CSD**	5.15 ± 0.18 ^{b}	−0.51 ± 0.25 ^{a}	6.80 ± 0.14 ^{b}	89 ± 8 ^{a}	0.20 ± 0.02 ^{a}	0.03 ± 0.0 ^{b}	4.33	(4.73 ± 0.32) × $10^{8\,a}$	(7.20 ± 0.44) × $10^{7\,a}$
**CBY**	Nd	nd	7.58 ± 2.79 ^{b}	72 ± 9 ^{a}	<0.01	nd			(7.00 ± 0.10) × $10^{7\,a}$

SD: dough with sourdough and only wheat flour; CSD: dough with sourdough and made of wheat flour and cricket powder; CBY: dough with baker's yeast and made of wheat flour and cricket powder.Values in the same column with different letters (a–b) are significantly different ($p < 0.05$). nd: not determined.
